# An R package for ensemble learning stacking

**DOI:** 10.1093/bioadv/vbad139

**Published:** 2023-09-29

**Authors:** Taichi Nukui, Akio Onogi

**Affiliations:** Department of Life Sciences, Faculty of Agriculture, Ryukoku University, Otsu, Shiga 520-2194, Japan; Department of Life Sciences, Faculty of Agriculture, Ryukoku University, Otsu, Shiga 520-2194, Japan

## Abstract

**Summary:**

Supervised learning is widely used in biology for prediction, and ensemble learning, including stacking, is a promising technique for increasing and stabilizing the prediction accuracy. In this study, we developed an R package for stacking. This package depends on the R package caret and can handle models supported by caret. Stacking involves cross-validation of training data with multiple base learners, and the predicted values are used as explanatory variables for the meta-learner. In the prediction, the testing data were fed into the base models, and the returned values were averaged for each base learner. The averaged values were then fed into the meta-model, and the final predictions were returned. Using this package, the training and prediction procedures for stacking can be conducted using one-row scripts.

**Availability and implementation:**

The R package stacking is available at the Comprehensive R Archive Network (CRAN) (https://cran.r-project.org/) and GitHub (https://github.com/Onogi/stacking). R scripts to reproduce the presented results are also reposited at GitHub.

## 1 Introduction

Ensemble learning is a promising approach to prediction tasks in biology (e.g. Sammut *et al.* 2022). Stacking is an ensemble learning method that can be applied to various biological processes. One application is genomic prediction, a statistical technique for predicting phenotypes/genetic merits using genome-wide DNA markers. Liang *et al.* (2021) showed that stacking using support vector regression, kernel ridge regression, and elastic nets as base learners had, on average, 7.70% higher prediction accuracy in three datasets than the genomic best linear unbiased prediction, a widely used method for genomic prediction. However, stacking requires cumbersome scripts. It also requires a longer computation time to train the models because multiple models should be trained.

We developed the R package “stacking” in this study to overcome these problems. This package is based on the R package “caret” (Kuhn 2008), which enables users to use various supervised learning methods in the same manner via the wrapper functions provided by the package. Users can choose any model supported by caret and conduct stacking without cumbersome scripting. The R package stacking implements parallel computations using the R package parallel to enable the parallel training of multiple learners. The advantages over the preceding package “stacks” which implements stacking (Couch *et al.* 2023) are that “stacking” covers more models implemented in caret and is implemented in non-tidyverse methods which will be familiar to users of machine learning packages similarly implemented (e.g. glmnet, Friedman *et al.* 2010).

## 2 Description

**Figure 1.**
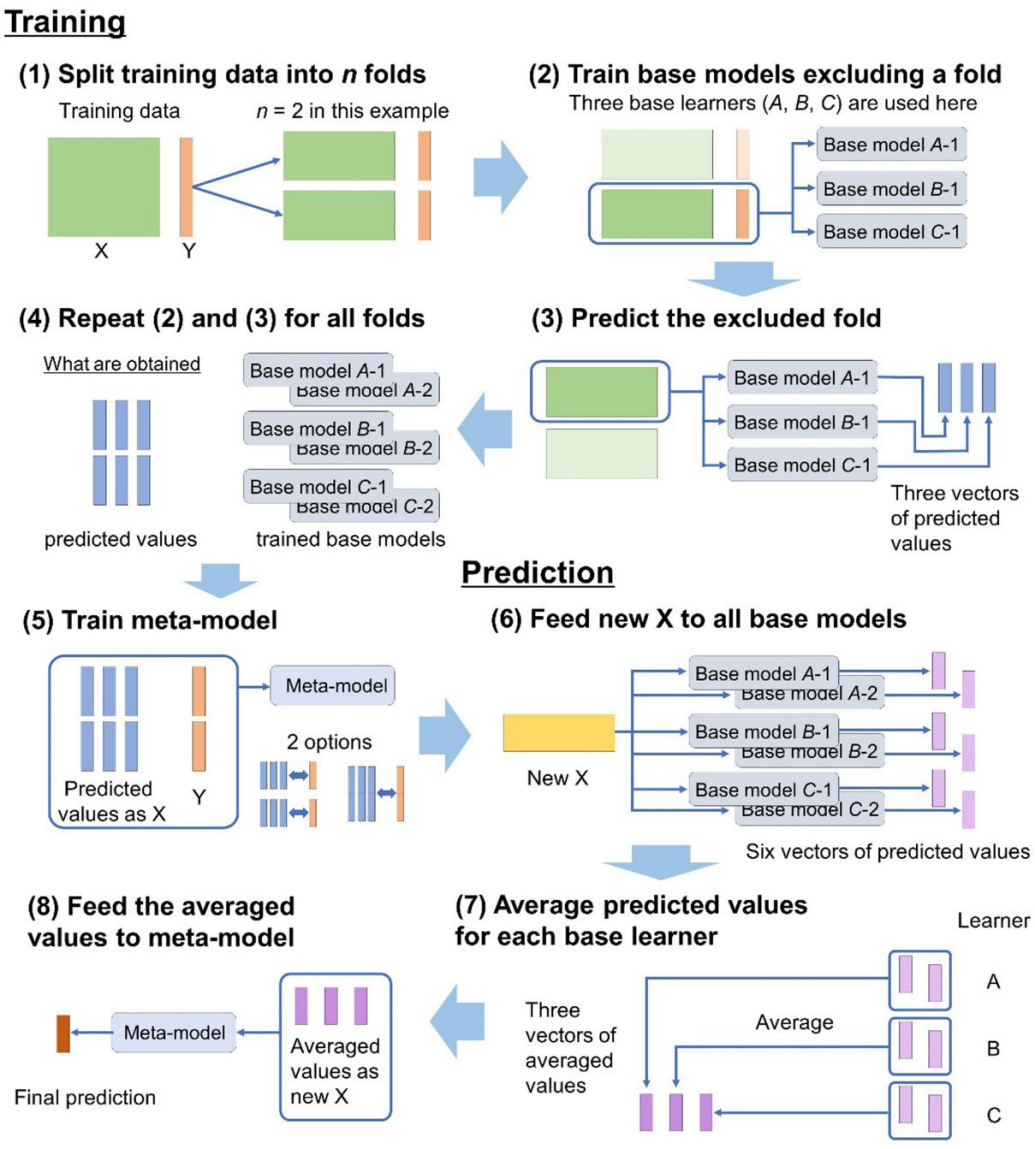
Implemented stacking strategy. In training, a cross-validation is conducted using each base learner (Steps 1–4), and the predicted values are used for training the meta-learner as the explanatory variables (Step 5). Here two options are available: training with all folds (right) and training within each fold (left). In prediction, new X is fed to all base models (Step 6), and the predicted values are averaged for each base learner (Step 7). The averaged values are then fed to the meta-model as the new explanatory variables (Step 8).

The stacking strategy implemented in our package is illustrated below using an example of regression tasks in which the number of cross-validation (CV) folds is two, and the number of base learners (members of the ensemble) is three. This strategy is illustrated in Fig. 1. First, a CV is conducted with each learner using the training data (Steps 1–4 in Fig. 1). Subsequently, a meta-learner is trained using the predicted values of each base model as explanatory variables (i.e. using three explanatory variables) (Step 5).

Because each base learner is trained two times in this training process, six (2 × 3) base models are built. The testing data is first provided to the base models in prediction, resulting in six predicted values (Step 6). The predicted values are averaged for each learner, resulting in three predicted values (Step 7). Using these values as explanatory variables for the meta-model, the final predicted values are obtained (Step 8). In classification tasks, the predicted values of the base models are not averaged. Instead, the most frequent categories are used as explanatory variables in the meta-model.

Two options were considered during the training of the meta-learner. First, the meta-learner is trained once using the predicted values pooled across folds as explanatory variables (to the right of the two options in Step 5 of Fig. 1). Second, the meta-learner is trained on the number of folds (two in Fig. 1) using the predicted values of each fold separately (left of the two options). In the prediction of the latter option, the predicted values of each meta-model are averaged to obtain the final predictions. In the package “stacking,” these two options can be switched with an argument *TrainEachFold*. The default setting is *TrainEachFold* = FALSE, indicating the former option. The following experiments used this option.

Package stacking primarily comprises four functions: *stacking_train*, *train_basemodel*, *train_metamodel*, and *stacking_predict*. *stacking_train* and *stacking_predict* are functions for training and prediction, respectively. The former internally calls *train_basemodel* and *train_metamodel*, but users can also call these functions themselves to optimize the base and meta-learners. Because the training process of base learners can require a long computational time, *train_basemodel* implements parallel computation using the parallel package.

*stacking_train* takes seven arguments: *Y*, *X*, *Nfold*, *Method*, *Metamodel*, *TrainEachFold*, and *core*. *Y* and *X* are the vector and matrix of the objective and explanatory variables, respectively. *Nfold* is a scalar indicating the number of CV folds. *Method* is a list containing data frames as elements. The names of the elements specify the methods of the base learners and are passed on to the caret functions. Each element (i.e. the data frame) includes the hyperparameters of each learner. When the number of rows of the data frame is >1, i.e. when multiple hyperparameter values are given, all combinations of hyperparameter values are automatically created and treated as different base learners. *Metamodel* is a character that indicates the meta-learner. *TrainEachFold* determines the training approach of meta-model, as described above. *core* is a scalar that indicates the number of cores required for parallel computing.

*stacking_train* passes arguments *Y*, *X*, *Nfold*, *Method*, and *core* to *train_basemodel*; then, the list output by *train_basemodel* and arguments *Metamodel* and *TrainEachFold* are given to *train_metamodel*. *train_metamodel* also takes an additional argument, *which_to_use*, that indicates which base models are used for training the meta-model. *train_metamodel* then outputs the training results of the meta-model as a list, which is then output by *stacking_train*. *stacking_predict* function takes two arguments: *newX* and *stacking_train_result*. *newX* is a matrix containing the explanatory variables of the new data, and *stacking_train_result* is the list output by *stacking_train*. *stacking_predict* outputs a vector of the predicted values.

First, we demonstrate the use of stacking using simulations. An explanatory variable matrix (*X*) with 1000 samples and 200 explanatory variables was randomly generated from a standard normal distribution as the training data. A vector of objective variables (*Y*) was then created by setting the regression coefficient of the first 20 explanatory variables to one and the remaining variables to zero.

**Figure 2.**
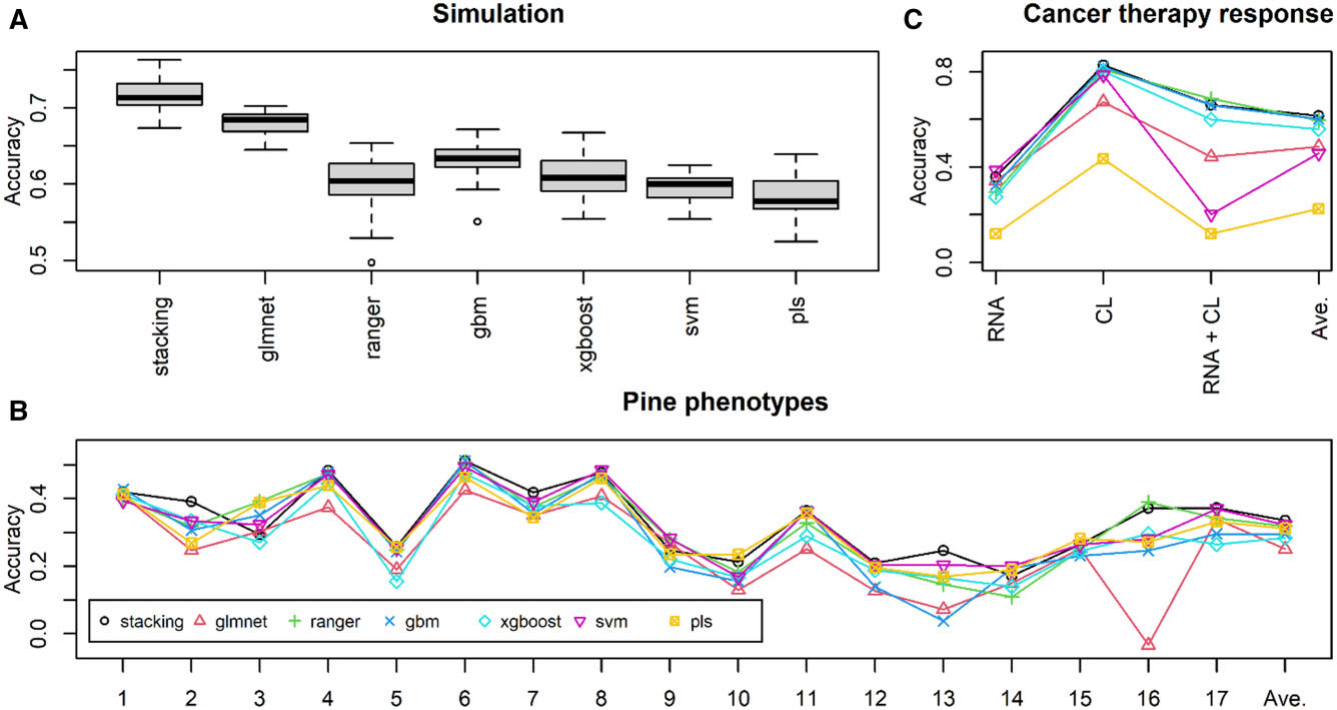
(A) Prediction accuracy evaluated with the Pearson correlation coefficients in simulation analyses. The number of replicates was 20. (B) Prediction accuracy evaluated with the Pearson correlation coefficients in the loblolly pine data analyses. The numbers on the *x*-axis indicate traits. The trait abbreviations are 1, HTLC; 2, BA; 3, BD; 4, BLC; 5, C5C6; 6, CWAS; 7, CWAL; 8, DBH; 9, Density; 10, Gall; 11, HT; 12, LateWood.4; 13, Lignin; 14, Rootnum; 15, Rootnumbin; 16, Rustbin; and 17, StiffnessTree. See Resende *et al.* (2012) for the descriptions of these traits. (C) Prediction accuracy evaluated with the kappa coefficients in the cancer therapy data analyses. CL and RNA denote clinical and RNA features, respectively.

Interactions between neighboring variables (i.e. between first and second, second and third, and so on) were also considered by multiplying the corresponding variables and giving regression coefficients of one. Random noise was then added such that the signal-to-noise ratio was four. Testing data of the same size were similarly generated in the same manner. As the base learners, major supervised learning methods including random forests implemented by ranger ver. 0.14.1 or 0.15.1 (Wright and Ziegler 2017), boosting by xgboost ver. 1.7.5.1 (Chen and Guestrin 2016), and gbm ver. 2.1.8.1 (Greg 2007), support vector machine with a radial basis function (RBF) kernel by kernlab ver. 0.9-31 or 0.9-32 (Karatzoglou *et al.* 2004), elastic net by glmnet ver. 4.1-6 or 4.1-7, and partial least squares regression by pls ver. 2.8-1 (Mevik and Wehrens 2015) were used. We used caret ver. 6.0-93 or 6.0-94 on R ver. 4.2.2 or 4.2.3, respectively. The numbers of hyperparameter sets were nine for ranger, eight for xgboost, nine for gbm, nine for support vector machine, six for glmnet, and seven for pls, resulting in 48 base learners. The hyperparameters to be specified can be confirmed using the *modelLookup* function of caret, and plausible values can be confirmed by applying methods to the data with caret. As the meta-model, elastic net implemented by glmnet was used. The training of stacking can be executed, e.g. using the script:

*stacking_train* (*X.train*, *Y.train*, *5*, *Method*, *Metamodel*, *4*)

where *X.train* and *Y.train* are the explanatory and objective variables of the training data, respectively, *5* is the number of CV, *Method* is the list specifying the base learners and their hyperparameters, *Metamodel* specifies the meta-learner, and *4* is the number of cores used for parallel computation. The prediction accuracy, which was evaluated as the Pearson correlation coefficient between the predicted and true values, is shown in Fig. 2A. Here, stacking was compared with the methods used as base learners. These methods compared were executed through caret. Stacking showed the best accuracy on average.

Next, we used data from loblolly pine (Resende *et al.* 2012) as an example of a regression task. The explanatory variables were the genotypes of 4853 SNPs, and the objective variables were the phenotypes of 17 traits. The number of samples was 926: 20% of samples were randomly assigned to testing data, and the remaining 80% were used for training. The base learners and hyperparameters of stacking were the same as those in the simulation study, except for *mtry* of ranger and *ncomp* of pls, which were modified according to the data size. The meta-model was also the same as that in the simulation study (elastic net). The prediction accuracy, evaluated using the Pearson correlation coefficient, is shown in Fig. 2B, where stacking exhibits the best accuracy on average. Stacking showed the best or nearly the best accuracy for most traits, whereas the rankings of the compared methods fluctuated across traits.

We then used data from breast cancer therapy as an example of a classification task (Sammut *et al.* 2022). The objective variable was a response to neoadjuvant treatment, which consisted of four categories: pCR, RCB-I, RCB-II, and RCB-III. pCR denotes no tumor, and the remaining indicates tumor residuals, in which the magnitude worsens as the number increases. To predict the response, multiple types of information, such as clinical features (e.g. tumor grade), RNA features (amounts of transcripts), and DNA features (e.g. mutation burden), were collected from the patients. Here, clinical and RNA features were used as explanatory variables because of their significant contribution to the response. Before evaluation, the top 2000 transcripts from 57 905 transcripts were selected based on *P*-values obtained with Wilcoxon rank-sum tests between pCR and other categories to reduce computational time. The base learners used for the regression example above were also used, except for the RBF kernel of support vector machine was replaced with a polynomial kernel because the RBF kernel often failed to predict minor categories. Random forests implemented by ranger were chosen as the meta-learner. The prediction accuracy was evaluated using a 5-fold CV. The kappa coefficient was used as the metric to evaluate the accuracy, which was calculated using the R package epiR (Carstensen *et al.* 2022). The kappa coefficients between the observed and predicted categories are shown in Fig. 2C, where stacking also showed the best accuracy on average and stable performance in terms of ranking among the methods, irrespective of the explanatory variables used.

## 3 Conclusions

Package stacking enables users to conduct stacking without cumbersome scripts. The experiments presented show that stacking can make accurate and robust predictions, irrespective of the type of tasks and explanatory variables.
